# Long Non-Coding RNA Profiling in Laryngeal Squamous Cell Carcinoma and Its Clinical Significance: Potential Biomarkers for LSCC

**DOI:** 10.1371/journal.pone.0108237

**Published:** 2014-09-22

**Authors:** Zhisen Shen, Qun Li, Hongxia Deng, Dakai Lu, Haojun Song, Junming Guo

**Affiliations:** 1 Department of Otorhinolaryngology (Head and Neck Surgery), Lihuili Hospital of Ningbo University, Ningbo, China; 2 Ningbo University School of Medicine, Zhejiang Provincial Key Laboratory of Pathophysiology, Ningbo, China; Karolinska Institutet, Sweden

## Abstract

Long non-coding RNAs (lncRNAs) are novel transcripts that may play important roles in cancer. Our study aimed to resolve the lncRNA profile of larynx squamous cell carcinoma (LSCC) and to determine its clinical significance. The global lncRNA expression profile in LSCC tissues was measured by lncRNA microarray. Distinctly expressed lncRNAs were identified and levels of AC026166.2-001 and RP11-169D4.1-001 lncRNAs in 87 LSCC samples and paired adjacent normal tissue were analyzed by real-time quantitative reverse transcriptase-polymerase chain reaction (qRT-PCR). The clinical significance of these lncRNAs in laryngeal cancer was analyzed and survival data were estimated by the Kaplan–Meier method and the log-rank test. A receiver operating characteristic (ROC) curve was constructed to check the diagnostic value. In the lncRNA expression profile of tumor samples, 684 lncRNAs were upregulated and 747 lncRNAs were downregulated (fold-change >2.0). Of these, AC026166.2-001 and RP11-169D4.1-001 were distinctly dysregulated, with AC026166.2-001 exhibiting lower expression in cancer tissues and RP11-169D4.1-001 higher expression. We verified that both AC026166.2-001 and RP11-169D4.1-001 were expressed at a lower level in cervical lymph nodes compared with paired laryngeal cancer tissues and paired normal tissues. RP11-169D4.1-001 levels were positively correlated with lymph node metastasis (*P* = 0.007). From the survival analysis, decreased levels of AC026166.2-001 and RP11-169D4.1-001 were associated with poorer prognosis. The area under the ROC curve was up to 0.65 and 0.67, respectively, and the cut-off point of ΔCt was 11.23 and 10.53, respectively. AC026166.2-001 and RP11-169D4.1-001 may act as novel biomarkers in LSCC and may be potential therapeutic targets for LSCC patients. Both AC026166.2-001 and RP11-169D4.1-001 could be independent prognostic factors for survival in LSCC.

## Introduction

Laryngeal cancer, one of the most common malignancies in the head and neck region, is the 11^th^ most common malignancy in males [1]. More than 95% of laryngeal cancers are laryngeal squamous cell carcinomas (LSCC), and are rarely adenocarcinomas [2]. Most LSCC are glottis (>60%) and supraglottis cases, with the subglottis representing the minority of patients (<5%) [2]. Patients with invasion and metastasis of laryngeal carcinoma have much worse prognosis, with a 5-year survival rate of approximately 60% [1]. In recent years, surgery and radiation methods with or without chemotherapy have been utilized as the main treatments for LSCC, but the majority of these have devastating results on swallowing or speaking functions. During the early stages of LSCC, there are few typical symptoms. The basic diagnostic methods for laryngeal cancer are computerized tomography (CT), magnetic resonance imaging (MRI) and histopathological examination through laryngoscopy. However, there is a lack of sensitive and specific biomarkers for early diagnosis, and laryngeal cancer is complex and poorly understood. Thus, identification of the molecular mechanisms of LSCC is critical to improving diagnosis and treatment.

Mammalian genomes can be transcribed into vast numbers of transcripts. Aberrant expression of transcripts, including those of protein-coding RNA and non-protein RNA, results in cancer [3]. Early studies identified many aberrantly expressed protein-coding genes, many of which are well understood. Recently, researchers have mainly focused on non-coding RNAs (ncRNAs), which lack protein-coding potential. Small ncRNAs and mid-size ncRNAs, such as microRNAs (miRNAs) and Piwi-interacting RNAs (piRNAs), which are defined as less than 200 nucleotides (nt), have occupied the bulk of studies on ncRNAs. Lately, long non-coding RNAs (lncRNAs), more than 200 nt in length, have gained increasing attention in cancer research. LncRNAs can be divided into several groups according to their relationship with protein-coding genes: sense lncRNAs, antisense lncRNAs, bidirectional lncRNAs, intronic lncRNAs and intergenic lncRNAs [4]. Recent reports have shown that lncRNAs play important roles at both the transcriptional and post-transcriptional level [5]. Furthermore, lncRNAs possess a myriad of functions such as chromatin remodeling, RNA decay, epigenetic regulation and chromatin modification [6], [7]. A growing number of lncRNAs have been shown to participate in various disease processes. For example, taurine-upregulated 1 (TUG1), nuclear paraspeckle assembly transcript 1 (NEAT1) and maternally expressed 3 (MEG3) are deregulated in neurodegenerative disorders [8]. Imprinted maternally expressed transcript-H19, X inactive specific transcript (XIST) and long noncoding RNA associated with liver regeneration 1 (lncRNA-LALR1) were noted in liver pathophysiology [9]. The importance of lncRNAs in several cancer types, including prostate cancer, breast cancer, gastric cancer, liver cancer and glioma have also been addressed [8]–[10]. Reports revealed that lncRNAs can function as tumor suppressors or oncogenes in various cancers, and are involved in tumor cell proliferation, migration and metastasis [11]. However, their detailed mechanisms are still unclear.

The contribution of lncRNAs in laryngeal cancer remains largely unknown, and no fully global lncRNA expression profile for LSCC was available. In this study, an lncRNA expression profile was established from seven paired LSCC specimens and adjacent normal tissues through microarray platform. Five lncRNAs were randomly selected from the top 20 upregulated and top 20 downregulated lncRNAs. According to their fold-change, *P*-value, length and sequence, primer specificity and pre-test results, two lncRNAs, AC026166.2-001 and RP11-169D4.1-001, were selected for further study. Finally, we focused our attention on the expression pattern of these representative lncRNAs in LSCC tissues and determined whether they could be biomarkers for LSCC. We also analyzed the relationship between their expression levels and clinicopathological features.

## Materials and Methods

### Patient sample collection

In this study, laryngeal cancer patients who had not received prior radiotherapy, chemotherapy and other antitumor treatment were recruited during January 2009 to February 2010. Matched tumor samples, non-cancerous tissues and metastatic neck lymph nodes were obtained during surgery at the Department of Otolaryngological, Ningbo Lihuili Hospital and clinicopathological data was collated. Non-cancerous tissues were excised at 1.5 cm from the tumor-free margin. All LSCC samples were examined by two or more independent pathologists. Tumor stages were determined according to the American Joint Committee on Cancer (AJCC) tumor-node-metastasis (TNM) staging criteria. This study was approved by the Human Research Ethics Committee of Ningbo University. Written informed consent was obtained from all subjects. Each sample was stored in RNA Fixer Reagent (Bioteke, Beijing, China) at −80°C until use.

### Total RNA preparation

To isolate total RNA, the frozen tissues were minced with a homogenizer (IKA, Staufen, Germany) and extracted using TRIzol reagent (Invitrogen, Carlsbad, CA). The samples were quantified and assessed for quality with a Nanodrop and Agilent 2100 Bioanalyzer (Agilent Technologies, Palo Alto, CA), respectively. The purity of total RNA was examined by the absorbance ratio at 260 to 280 nm [12].

### LncRNA expression analysis

Seven paired tissue specimens were obtained from LSCC patients with different clinical stages and differentiated pathology (Table 1). After purification, RNA was amplified and transcribed into cDNA. Then the cDNA was labeled and homogenized according to the manufacturer’s protocol. An Agilent Quick Amp Labeling Kit (Agilent Technologies, Santa Clara, CA) was used to label 1µg of total RNA, and an Agilent Gene Expression Hybridization Kit (Agilent p/n 5188-5242) was used for hybridization. Hybridization was performed in a SureHyb Hybridization Chamber (Agilent) at 65°C for 17 h. Agilent Array platform was employed for microarray analysis. After washing, the slides were scanned with an Agilent DNA Microarray Scanner (Agilent p/n G2565BA), and data were extracted using Agilent Feature Extraction software. Further data analysis was performed using Agilent GeneSpring GX v12.0 software. The Human LncRNA Microarray V2.0 (Arraystar) contains more than 33,000 lncRNAs. The data for the lncRNA microarray are collected from authoritative databases, such as National Center for Biotechnology Information (NCBI) RefSeq, Ensemble database, University of California, Santa Cruz (UCSC), lncRNA database (lncRNAdb), lncRNAs from published literature and Ultra Conserved Regions (UCRs). The microarray work was performed by KangChen Bio-tech, Shanghai, China.

**Table 1:** Clinical parameters of seven laryngeal cancer patients that underwent lncRNA expression profiling.

Specimen No.	Histologic differentiation	Age (years)	TNM Stage
No. 1	Moderately	61	T3N1M0
No. 2	Well	54	T2N1M0
No. 3	Poorly	54	T3N1M0
No. 4	Moderately	53	T3N2M0
No. 5	Dysplasia	77	Tis
No. 6	Dysplasia	66	Tis
No.7	Moderately	58	T1N0M0

### Reverse transcription reaction and quantitative real-time RT-PCR

cDNA was reverse transcribed using the GoScript Reverse Transcription (RT) System (Promega, Madison, WI) according to the manufacturer’s instructions. LncRNA expression levels in the samples were calculated using the threshold cycle (Ct) method and normalized to the reference control gene *GAPDH* [13]. Higher ΔCt values corresponded to lower expression levels. All primers were synthesized by Invitrogen (Shanghai, China). The primer sequences were as follows: *GAPDH*: sense, 5′-ACCCACTCCTCCACCTTTGAC-3′; antisense, 5′-TGTTGCTGTAGCCAAATTCGTT-3′; *AC026166.2-001*: sense, 5′-CCACGATGTTTCCTTGGTTTA-3′; antisense: 5’- ATTTGTGATTGAAAGGTTCTACTAC-3′; *RP11-169D4.1-001*: sense, 5′-TCTCACTAAGGTAGAACTGATGGGC-3′; antisense, 5′-GACTCCTCAGGGAAAATGGAAACT-3′. The real-time quantitative reverse transcriptase-polymerase chain reaction (qRT-PCR) conditions for lncRNAs were as follows: 10 min at 95°C, followed by 45 cycles at 95°C for 15 s, 56°C for 30 s, and 72°C for 30 s. Reactions were performed with 5 µl cDNA according to the standard protocol for GoTaq qPCR master mix (Promega, Madison, WI). All the samples were assayed in triplicate.

### Statistical analysis

SPSS software 18.0 (SPSS Inc., Chicago, IL) was used to analyze the data. Expression levels between LSCC cancer tissues and adjacent non-tumor tissues were analyzed by paired-sample *t*-tests. The correlation between expression levels and clinicopathological factors was further analyzed with independent-sample *t*-tests. *P*-values below 0.05 were regarded as statistically significant. The diagnostic value was evaluated with a receiver operating characteristic (ROC) curve. The Kaplan–Meier method was used to compare the survival data between the different expression level markers.

## Results

### Patient characteristics

Eighty-seven patients were recruited to the study and followed up for 48 months. The median age was 62 years (range, 42–82 years), and the majority of subjects consisted of males (95%). Thirty-four patients (39%) were diagnosed with neck lymph nodal metastasis (Table 1).

### Overview of lncRNA profile

**Figure 1:**
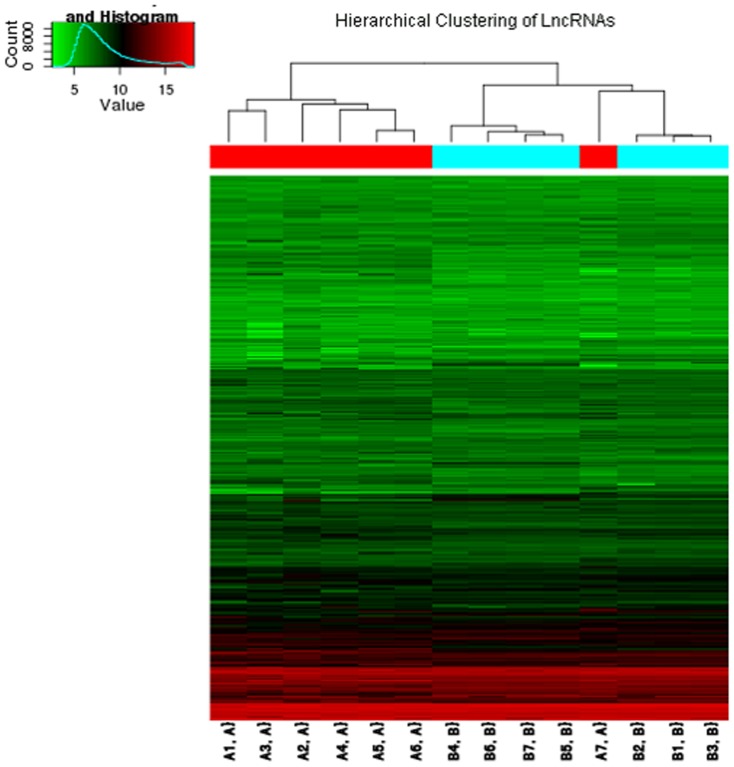
Hierarchical clustering results of lncRNA expression profiles in LSCC samples and adjacent normal tissue. Distinct differences were observed between LSCC tissue and adjacent non-tumorous tissue. “A”, tumor group; and “B”, normal group.

**Figure 2:**
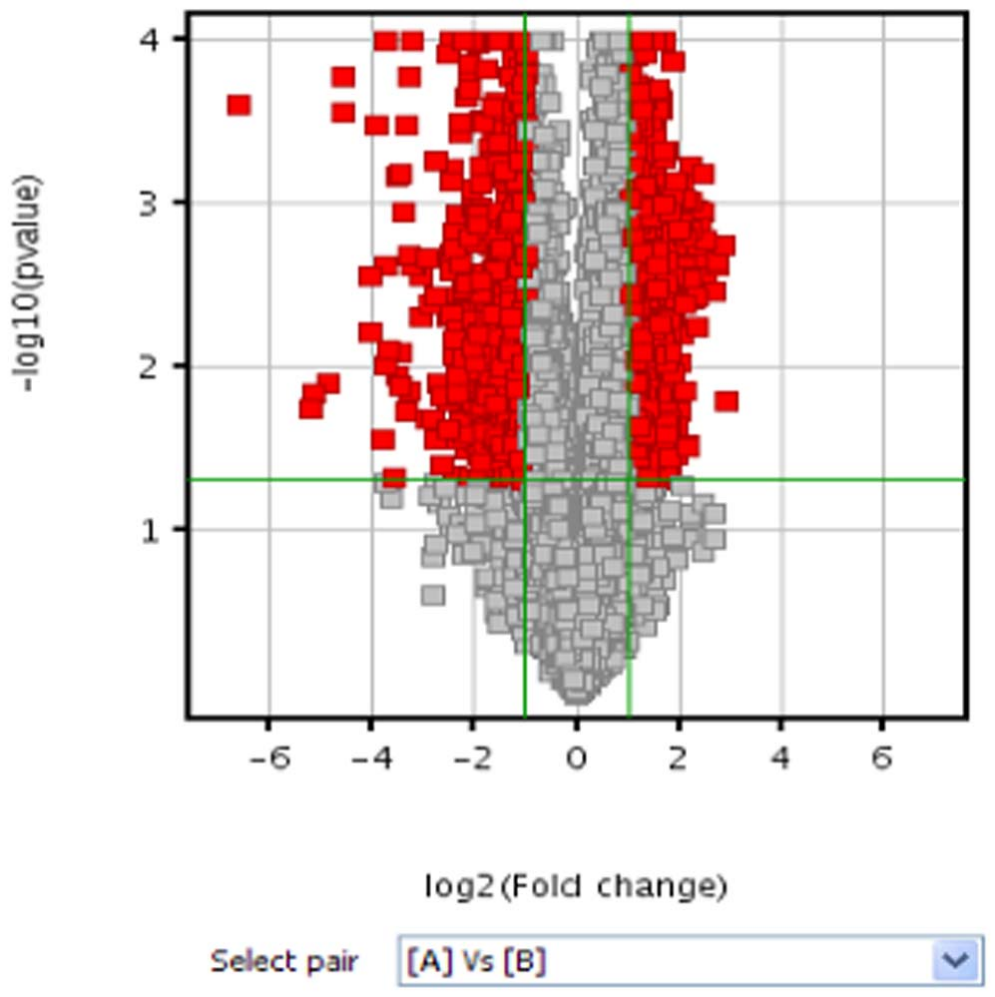
Volcano plot filtering map depicting differentially expressed lncRNAs. Red points represent differentially expressed lncRNAs with statistical significance (P<0.05). A and B represent cancer and normal, respectively.

We conducted lncRNA profiling between laryngeal cancer and paired normal tissue samples. Up to 33,045 lncRNAs probes were used to detect lncRNAs in seven pairs of LSCC tissues. Distinct differences were found between LSCC tissue and nearby non-tumorous tissues. Hierarchical clustering was performed, from which we concluded the expression patterns in LSCC tissue were significantly different from adjacent normal tissue (Figure 1). The microarray profile has been deposited in National Center for Biotechnology Information (NCBI) Gene Expression Omnibus (GEO). The accession number is GSE59652 (http://www.ncbi.nlm.nih.gov/geo/query/acc.cgi?acc=GSE59652). In this study, 684 lncRNAs were upregulated and 747 lncRNAs were downregulated in tumor samples (fold-change >2.0) (Table 2), and volcano plot filtering was used to identify the differentially expressed lncRNAs (Figure 2). The most upregulated lncRNAs in LSCC tissues were AK075442, BC045564, AF086267, AK057984, ENST00000513381, BM413624, ENST00000490139, HIT000342137, and ENST00000450804, while CR595807, uc001rml.1, uc003fah.1, ENST00000499050, NR_001284, ENST00000405071, AK093732, ENST00000510453, NR_001564, and uc001jkr.2 represented the most downregulated lncRNAs in LSCC samples.

**Table 2:** Differentially expressed lncRNAs in LSCC tissue compared with adjacent non-tumorous tissue.

Up-regulated in tumor tissues (*P*<0.01)			Down-regulated in tumor tissues (*P*<0.01)		
lncRNAs	Fold change	Source	lncRNAs	Fold change	Source
AK075442	7.41	misc_RNA	CR595807	99.79	misc_RNA
BC045564	7.17	misc_RNA	uc001rml.1	37.26	UCSC_knowngene
AF087976	6.63	misc_RNA	NR_001284	24.30	RefSeq_NR
ENST00000513381	6.26	Ensembl	ENST00000433573	13.01	Ensembl
ENST00000450804	5.26	Ensembl	ENST00000499050	9.01	Ensembl
uc.162-	5.17	uc.162	ENST00000369884	8.38	Ensembl
G36445	4.67	misc_RNA	NR_028130	7.04	RefSeq_NR
AK025038	4.63	NRED	NR_026756	6.32	RefSeq_NR
HIT000324184_03	3.85	H-invDB	BC047057	5.73	misc_RNA
DB351988	3.80	lincRNA-RPS24-2	BC043564	4.06	lincRNA
AK023033	2.86	RNAdb	NR_003670	3.88	RefSeq_NR
uc004elg.2	2.57	UCSC_knowngene	uc001gch.1	2.68	UCSC_knowngene

RefSeq_NR: RefSeq validated non-coding RNA; UCSC_knowngene: (http://genome.ucsc.edu/cgi-bin/hgTables/); Ensembl: (http://www.ensembl.org/index.html); H-invDB:(http://www.h-invitational.jp/); RNAdb:(http://research.imb.uq.edu.au/rnadb/); NRED:(http://jsm-research.imb.uq.edu.au/nred/cgi-bin/ncrnadb.pl); UCR:(http://users.soe.ucsc.edu/~jill/ultra.html); lncRNAdb:(http://www.lncrnadb.org/); misc_lncRNA: other sources.

AC026166.2-001 (Ensembl: ENST00000433573) was downregulated by 13.01-fold in LSCC tissues (*P* = 0.008). Conversely, RP11-169D4.1-001 (Ensemble: ENST00000450804) was upregulated by 5.26-fold in LSCC tissues (*P* = 0.002). Furthermore, AC026166.2-001 was downregulated and RP11-169D4.1-001 was upregulated in 6/7 paired specimens.

### AC026166.2-001 was downregulated in LSCC tissues and metastatic neck lymph nodes

Based on the profile data, AC026166.2-001 was found to be downregulated in six cancer sample tissues. We sought to identify if its expression levels correlated with clinicopathological features and if AC026166.2-001 could function as a molecular marker in laryngeal cancer.

**Figure 3:**
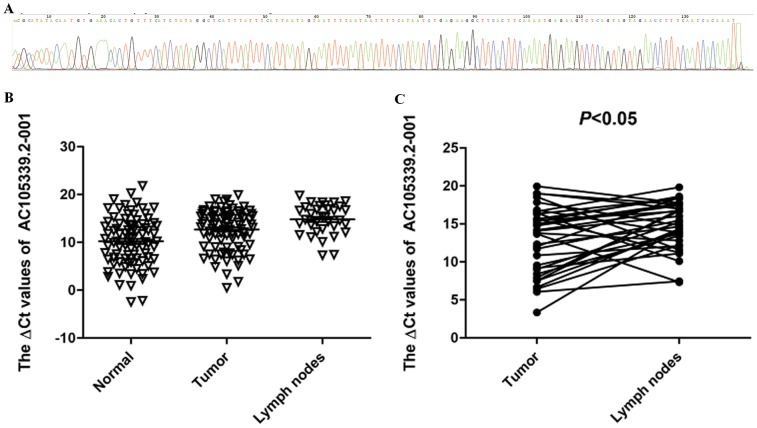
The expression of AC026166.2-001 by quantitative RT-PCR assay. (A) Sequencing results of AC026166.2-001. (B) Expression levels of AC026166.2-001 in LSCC tumor, adjacent normal samples (n = 87) and metastatic neck lymph nodes samples (n = 34). Real-time qRT-PCR was performed to determine the expression levels of AC026166.2-001. AC026166.2-001 levels were significantly lower in tumor tissues than in corresponding non-tumorous tissues (n = 87, P<0.001). (C) AC026166.2-001 expression was significantly lower in metastatic neck lymph nodes compared with matched tumor tissues (n = 34, P<0.05).

To verify the microarray result, 87 paired surgical samples including metastatic neck lymph nodes were collected to explore the expression levels of AC026166.2-001 by qRT-PCR. PCR sequencing confirmed the PCR product sequence (Figure 3A), which was inputted into the NCBI nucleotide Blast program and the Ensembl database, resulting in an accordance rate of 100%. AC026166.2-001 expression levels in 64 (73.6%) of 87 cancer tissues from patients with LSCC were significantly lower than those in corresponding normal tissues: (12.62±4.40 versus 10.21±5.04, respectively, *P*<0.001; Figure. 3B). Lymph node metastasis is a prognostic factor for LSCC. We observed that AC026166.2-001 expression was significantly lower in metastatic neck lymph nodes compared with matched normal tissues (*P*<0.001) and tumor tissues (*P*<0.05, Figure 3C).

As shown in Table 3, there was no significant different between AC026166.2-001 levels and age, tumor location, pathological type, and lymphatic metastasis. Smoking history is significantly related to laryngeal cancer [14], but there no statistical relationship between the expression level of AC026166.2-001 and smoking history could be identified in this study (*P* = 0.596).

**Table 3:** Relationship between AC026166.2-001 or RP11-169D4.1-001 levels (ΔCt) and clinicopathological features of laryngeal cancer patients.

Characteristics	No. of patients (%)	AC026166.2-001 (Mean±SD)	*P* value	RP11-169D4.1-001 (Mean±SD)	*P* value
Age (y)			0.564		0.852
≤60	39(44.8%)	12.93±4.06		10.47±4.05	
>60	48(55.2%)	12.37±4.69		10.31±4.12	
Primary location			0.547		0.545
Supraglottic	25(28.7%)	12.17±4.36		9.96±3.77	
Glottic	62(71.3%)	12.80±4.43		10.55±4.19	
Differentiation			0.514		0.640
Well & moderate	58(66.7%)	12.52±4.19		10.23±3.97	
poor	29(33.3%)	12.82±4.87		10.67±4.30	
Lymphatic metastasis			0.145		0.007
N0	53(60.9%)	12.61±4.05		9.45±4.21	
N1&N2&N3	34(39.1%)	12.64±4.96		11.83±3.40	
Invasion			0.742		0.991
T1-T2	55(63.2%)	12.75±4.02		8.17±3.89	
T3-T4	32(36.8%)	12.40±5.05		7.63±4.49	
Clinical stage			0.881		0.581
I-II	44(50.6%)	12.69±4.32		10.14±3.76	
III-IV	43(49.4%)	12.54±4.54		10.62±4.38	
Smoking history			0.697		0.823
Yes	68(78.2%)	12.72±4.41		10.33±4.13	
No	19(21.8%)	12.27±4.46		10.56±3.92	

### RP11-169D4.1-001 was downregulated in LSCC tissues and metastatic neck lymph nodes

**Figure 4:**
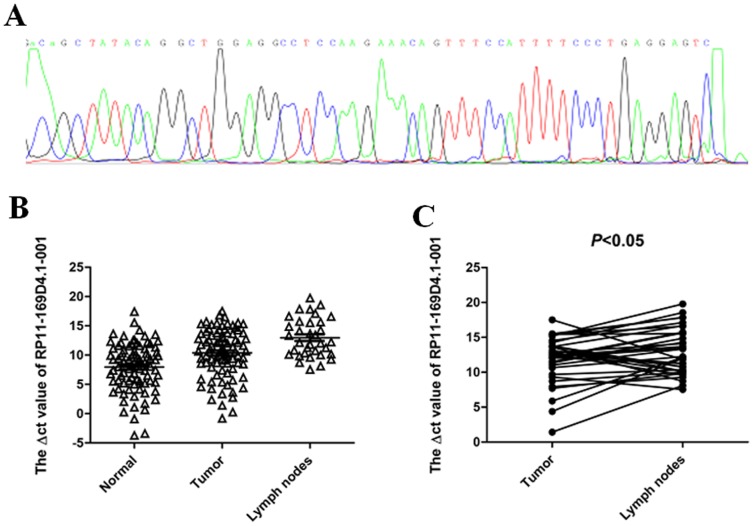
The expression of RP11-169D4.1-001 by quantitative RT-PCR assay. (A) RP11-169D4.1-001 expression levels in LSCC tumor, adjacent normal samples (n = 87) and metastatic neck lymph nodes samples (n = 34). Real-time qRT-PCR was employed to determine RP11-169D4.1-001 expression. RP11-169D4.1-001 levels were significantly lower in tumor tissues than in corresponding non-tumorous tissues (n = 87, P<0.001). (B) RP11-169D4.1-001 expression was significantly lower in metastatic neck lymph nodes compared with matched tumor tissues (n = 34, P<0.05). (C) Sequencing results for RP11-169D4.1-001.

RP11-169D4.1-001 was found to be upregulated in 86% (6/7) specimens from the microarray result. However, contrary to the profiling result, RP11-169D4.1-001 expression was lower in 77% (66/87) tumor tissues. The PCR product sequencing result was consistent with that from the Ensemble database (Figure 4A). We found RP11-169D4.1-001 was significantly downregulated in the tumor tissues compared with paired non-neoplastic tissues (10.38±4.06 versus 7.97±4.10, *P*<0.001; Figure: 4B,). Meanwhile, RP11-169D4.1-001 expression was downregulated in metastatic neck lymph nodes compared with paired tumor tissues (*P*<0.05; Figure 4C). Lymph node metastasis is an important factor for prognosis, therefore we analyzed whether the expression levels of RP11-169D4.1-001 in tumor tissues were associated with neck lymph node metastasis. As shown in Table 3, we observed significant differences in RP11-169D4.1-001 levels between patients with lymph node metastasis and without (*P* = 0.007). No significant differences were detected between other clinicopathological characteristics.

### Survival analysis

**Figure 5:**
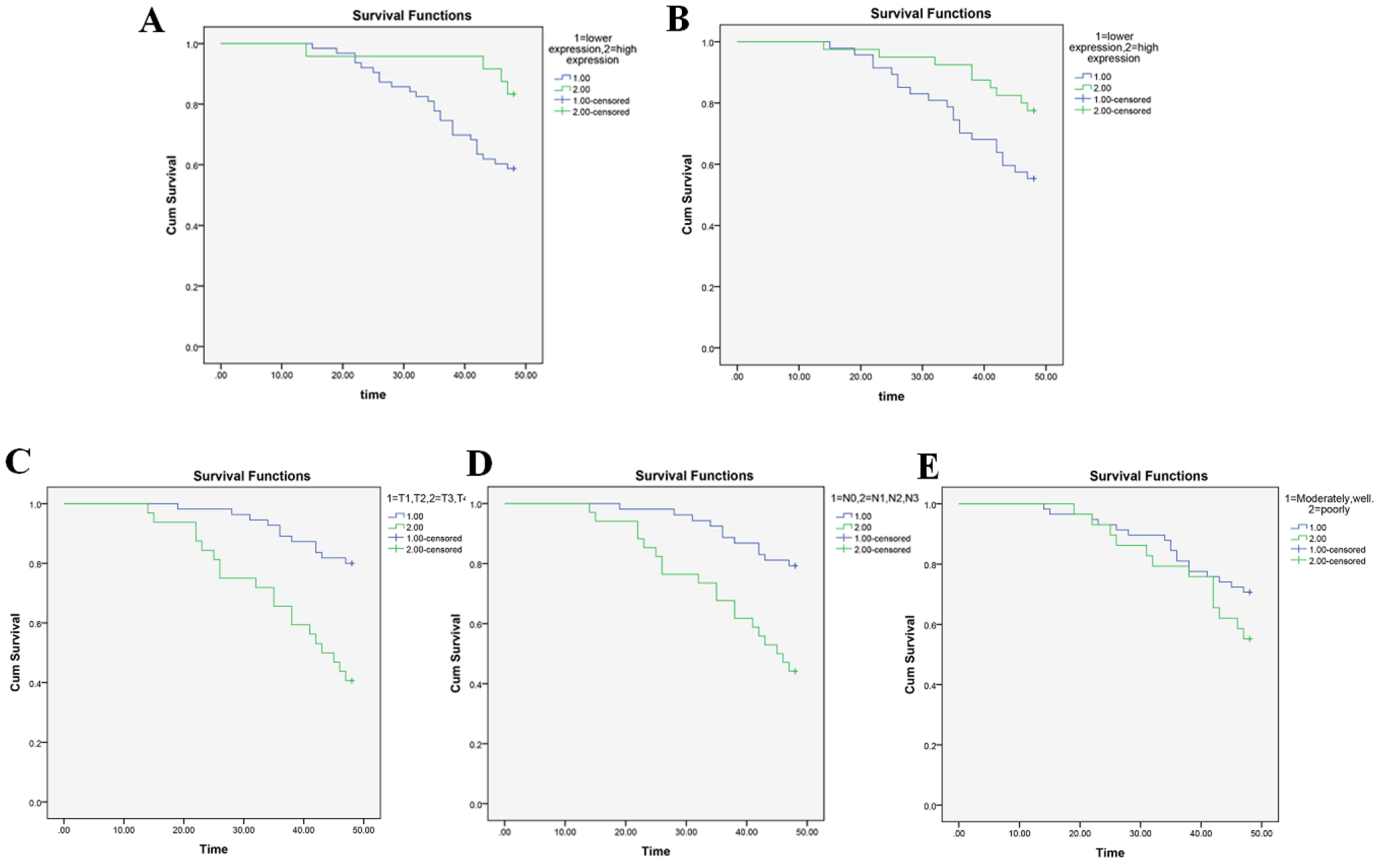
Survival curve of laryngeal carcinoma patients. (A) Survival curve of laryngeal carcinoma patients with varying expression levels of AC026166.2-001 (P = 0.029). Lower curve: LSCC patients with low expression (n = 63); upper curve: LSCC patients with high expression (n = 24). (B) Survival curves of laryngeal carcinoma patients (n = 87) with differing. RP11-169D4.1-001 expression levels (P = 0.025). Lower curve: LSCC patients with low expression (n = 47); upper curve: LSCC patients with high expression (n = 30). (C) Survival curves of laryngeal carcinoma patients according to T stage (P<0.001). (D) Survival curves of laryngeal carcinoma patients with and without cervical lymph node metastasis (P<0.001). (E) Survival curves of laryngeal carcinoma patients according to histological differentiation (P = 0.182).

All 87 cases were followed up for four years. Four patients missed follow-up and were grouped as dead; the overall survival rate was 65.5%. Patients were divided into two groups according to the cut-off point of each lncRNA. The log-rank *P*-value of the difference in progression-free survival is presented in Figure 5. Our study demonstrated that lower levels of AC026166.2-001 and RP11-169D4.1-001 were significantly correlated with overall survival (*P*<0.05). Kaplan–Meier analysis demonstrated that T grade (Figure 5C) and neck lymph node metastasis (Figure 5D) were significantly associated with survival time (*P*<0.001). However, no significant associations were detected by histological differentiation (*P* = 0.182). The Cox proportional hazards model was used for multivariate survival analysis, which determined that the expression levels of AC026166.2-001 (*P* = 0.003, 95% CI = 1.705–14.544) and RP11-169D4.1-001 (*P* = 0.026, 95% CI = 1.115–5.791) were independent prognostic factors for survival of LSCC patients.

### Evaluation of AC026166.2-001 and RP11-169D4.1-001 as diagnostic markers for LSCC

**Figure 6:**
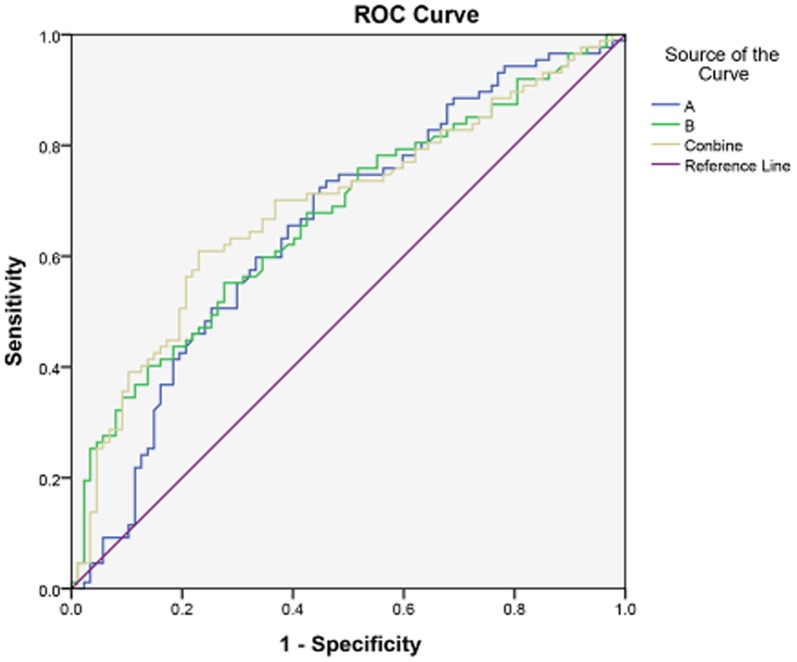
ROC curve. A represents AC026166.2-001, B represents RP11-169D4.1-001.

AC026166.2-001 and RP11-169D4.1-001 were detected in 64% and 77% of tumor samples, respectively. Furthermore, the areas under the ROC curve were 0.65 and 0.67, respectively (Figure 6), while the cut-off points of ΔCt was 11.23 and 10.53, respectively. The area under ROC curve for AC026166.2-001 and RP11-169D4.1-001 combined increased slightly to 0.69, inferring that combination of the two lncRNAs improved their diagnostic value.

## Discussion

Previously, expression profiling studies of LSCC mainly focused on mRNA and miRNA. In our previous reports, *DJ-1* [15], *HuR* [16] and miR-34a [13] were found to be associated with LSCC.

Tiling arrays and high-throughput sequencing technologies provide a wealth of data regarding ncRNAs, and genetic profiling has provided an increasing number of distinguishable expressed lncRNAs in many diseases [8], [17].

To date, many studies have explored the multiple functions of lncRNAs in cancer but it was unclear if lncRNAs have a role in laryngeal carcinoma progression. Previously, a study by Li et al. demonstrated that a lincRNA homeobox transcript antisense RNA (HOTAIR) was significantly overexpressed in LSCC tissue compared with normal tissue [18]. HOTAIR was involved in *PTEN* methylation in Hep-2 cells [18]. Moreover, the expression level of HOTAIR correlated with esophageal squamous cell carcinoma progression and prognosis. [19]–[21] Metastasis-associated lung adenocarcinoma transcript-1 (MALAT-1) has also been shown to be involved in the progression of LSCC [22]. Reis et al. has conducted a large-scale transcriptome analyses between nontumor and tumor tissues of head and neck cancers. They described 2,251 unspliced intronic lncRNAs expressed in head and neck tumors and being involved in transcriptional regulation. [23] In the current study, microarray techniques were used to compare the expression profiles of laryngeal cancer tissues and paired normal tissues; this is the first study to provide a complete lncRNA expression profile for LSCC. In total, more than 1400 lncRNAs were found to be differentially expressed by more than two-fold (*P*<0.05) between cancer tissues and normal tissues.

In our study, we chose two representative lncRNAs for further study and qRT-PCR was used to confirm the expression levels among 87 paired tissue samples. Differential expression levels of AC026166.2-001 and RP11-169D4.1-001 were identified from the lncRNA expression profiles. Both lncRNAs were abnormally expressed in LSCC tissues compared with adjacent normal tissues (*P*<0.05).

Pseudogenes are considered to arise from the mutation of transcripts or as a result of mistakes in transcription. Moreover, pseudogenes can be generated by the degeneration of protein-coding genes or integration of cDNA from reverse transcription, and can even be produced from other pseudogenes. Processed pseudogenes can be integrated by retrotransposition of mRNA [24]–[25]. The gene of AC026166.2-001 is a known processed pseudogene (ENSG00000233026). AC026166.2-001, a 344nt transcript, is an unspliced lncRNA expressed from the opposite strand within one intron of the gene SYN2. Many studies have demonstrated that pseudogenes are involved in the development of cancers, such as gastric cancer and non-small cell lung cancer [26]–[27]. Increasing lines of evidence have revealed that unspliced intronic sense lncRNAs possess important functions in regulating epigenetic targets and cell proliferation [28]–[29] However, the relationship between AC026166.2-001 and cancer remained unclear.

AC026166.2-001 was found to be significantly downregulated in 86% (6/7) of laryngeal cancer tissues studied compared with corresponding non-cancerous tissues, and qRT-PCR data were consistent with the expression profile. Furthermore, AC026166.2-001 expression was lower in metastatic cervical lymph nodes (Figure 3C). Unfortunately, no distinct differences were found between other clinical features examined. Survival data suggested the decreased expression of AC026166.2-001 in tumor tissues was associated with poor prognosis (*P*<0.05; Figure 5). Additionally, we constructed a ROC curve to evaluate the diagnosis values. The area under the ROC curve was 0.65 (Figure 6).

RP11-169D4.1-001 is a 1890 nt transcript transcribed from the intergenic regions of ENSG00000227467 gene (http://asia.ensembl.org/index.html), which is located on the forward strand of chromosome 11: 72,281,704–72,284,273. However, it is annotated as a protein-coding mRNA encoding a 125 amino acid long protein in GenBank. This putative peptide has no homolog in the NCBI database; and the transcript is probably a lincRNA. It is a little investigated lincRNA in laryngeal cancer and its biological functions and characteristic are undefined.

RP11-169D4.1-001 was upregulated in the tumor tissue among 86% (6/7) paired LSCC tissues in the microarray profile, while it was found to be downregulated in tumor tissues among 77% (66/87) paired LSCC samples (Figure 4B, *P*<0.001) by qRT-PCR analysis. This suggests that the profiling results among small samples can only be used as references and require further verification by qRT-PCR analysis of large samples. Metastasis always leads to poor prognosis in LSCC patients [30], and accompanied metastatic cervical nodes are the prognostic indicator for LSCC. Our results revealed that decreased RP11-169D4.1-001 expression levels in LSCC were significantly associated with cervical lymph node metastasis (Figure 4C, *P*<0.05), suggesting that RP11-169D4.1-001 may play critical roles not only in the tumorigenesis of LSCC but also in its migration. We concluded that RP11-169D4.1-001 could be a potential indicator to predict the metastasis of LSCC. Patients with lower levels of RP11-169D4.1-001 have poorer prognosis, and RP11-169D4.1-001 expression levels were significantly correlated with the survival time of laryngeal cancer patients (*P*<0.05; Figure 5B)

In conclusion, our data provides a comprehensive lncRNA profile for LSCC. Both AC026166.2-001 and RP11-169D4.1-001 were identified as novel lncRNAs with diagnostic value in LSCC, and thus could be potential therapeutic targets. More importantly, according to the survival data analysis, both may be independent factors for prognosis.
